# Spontaneous Bone Marrow Edema: Perfusion Abnormalities and Treatment with Surgical Decompression

**DOI:** 10.3390/ijms24076761

**Published:** 2023-04-05

**Authors:** Jake Littman, Holly Gil, Roy Aaron

**Affiliations:** 1 Department of Orthopedic Surgery, Warren Alpert Medical School of Brown University, Providence, RI 02903, USA; 2 Department of Radiology, Warren Alpert Medical School of Brown University, Providence, RI 02903, USA

**Keywords:** MRI, pathophysiology, venous stasis, bone pain, surgical decompression, intraosseous hypertension

## Abstract

Bone marrow edema (BME), also termed bone marrow lesions, is a syndrome characterized by bone pain and the appearance of high signal intensity on T2 fat-suppressed and short tau inversion recovery (STIR) MRI sequences. BME can be related to trauma or a variety of non-traumatic diseases, and current treatment modalities include non-steroidal anti-inflammatory drugs (NSAIDS), bisphosphonates, denosumab, extracorporeal shockwave therapy (ESWT), the vasoactive prostacyclin analogue iloprost, and surgical decompression. Spontaneous BME is a subset that has been observed with no apparent causative conditions. It is most likely caused by venous outflow obstruction and intraosseous hypertension. These are mechanistically related to impaired perfusion and ischemia in several models of BME and are related to bone remodeling. The association of perfusion abnormalities and bone pain provides the pathophysiological rationale for surgical decompression. We present a case of spontaneous BME and a second case of spontaneous migratory BME treated with surgical decompression and demonstrate resolution of pain and the high signal intensity on MRI. This report provides an integration of the clinical syndrome, MR imaging characteristics, circulatory pathophysiology, and treatment. It draws upon several studies to suggest that both the bone pain and the MRI characteristics are related to venous stasis, and when circulatory pathologies are relieved by decompression or fenestration, both the bone pain and the MRI signal abnormalities resolve.

## 1. Introduction

Bone marrow edema (BME), also termed bone marrow lesions and, currently, edema-like marrow signal intensity, is a syndrome characterized by the sudden onset of bone pain and the MRI appearance of high signal intensity on T2 fat-suppressed (FS) and STIR MRI sequences characteristic of water density [1,2]. It may not be a single pathological entity but rather several pathologies with a common MRI appearance [1,3]. It is a nonspecific hallmark of several nontraumatic diseases and is associated with bone and ligament trauma, osteoarthritis (OA), avascular necrosis (AVN), infections, and transient osteoporosis with different clinical symptomatologies, varying prognoses, and perhaps different clinical significance in these conditions [3,4,5,6,7]. It is also seen with no obvious associative or causative conditions, in which cases it has been termed spontaneous BME and has been distinguished clinically from AVN [8]. In many cases, BME spontaneously resolves and no treatment is necessary. However, a subset of patients experience intense or prolonged pain for whom treatment is needed.

A less common form of spontaneous BME is migratory BME, in which spontaneous, asynchronous, high-intensity signals on T2 FS MRI are accompanied by pain in different locations in one bone, usually in the knee, or more rarely in several bones at different times. It is also known as intra-articular regional migratory osteoporosis [9]. Migratory BME has been associated with low bone mineral density [2,10]. A review of the world literature in 2008 revealed 63 cases, although the condition is probably underreported and the true prevalence is unknown [11].

At our practice, we treat large, persistently painful (over 8 weeks) spontaneous BME of the knee with surgical decompression. We also decompress BME associated with AVN and mild OA, but do not decompress advanced OA, AVN with subchondral fracture or collapse, lesions associated with trauma, or mild, spotty lesions associated with other conditions, since they can resolve quickly. The surgical protocol consists of 1 or 2 arthroscopically and fluoroscopically guided 4 mm decompression portals into regions of cancellous BME as described in the case reports. To eliminate risks of fracture, corticocancellous transition zones are avoided as entry portals. Following decompression, patients are allowed to weight-bear to tolerance and usually undergo partial weight-bearing with 2 crutches for 1 week. No surgical complications have been encountered.

## 2. Case Reports

**Figure 1:**
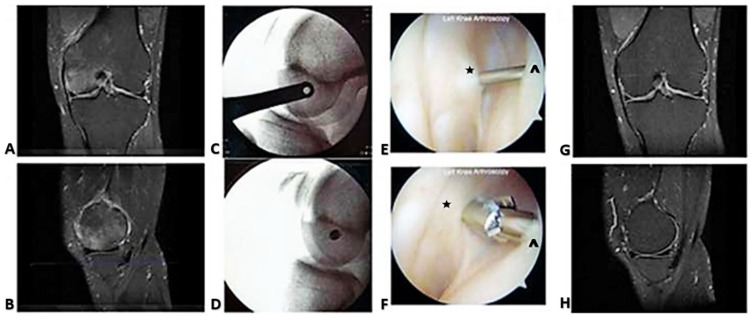
Surgical decompression of BME. (**A**) Coronal and (**B**) sagittal sections display the preoperative high signal intensity in the medial femoral condyle on the MRI T2 FS sequence. (**C**) Lateral X-ray views show an example of fluoroscopic guidance of pin placement, with the pin appearing as a dot (**D**), indicating parallelity with the fluoroscopic beam. (**E**, **F**) Arthroscopic guidance of pin entry and 4 mm cannulated drill tip through the joint capsule (*) to cancellous bone (^). (**G**) Coronal and (**H**) lateral MRI sections show resolution of the high signal intensity 3 months after decompression.

Case #1: A 60-year-old male with the spontaneous onset of disabling medial knee pain of 2 months duration. MRI demonstrated high signal intensity on T2 FS images at the medial femoral condyle (Figure 1). Under arthroscopic and fluoroscopic guidance, a guide pin was placed into the pathologic bone, taking care to remain proximal to the articular cartilage, and a 4 mm cannulated drill was used to create the surgical decompression. Pain relief occurred within 72 h. Follow-up MRI at 12 weeks post-decompression demonstrated resolution of the imaging abnormality and preservation of the structural integrity of the subchondral bone. At 1 year postoperative, the patient remained asymptomatic with normal knee function.

**Figure 2:**
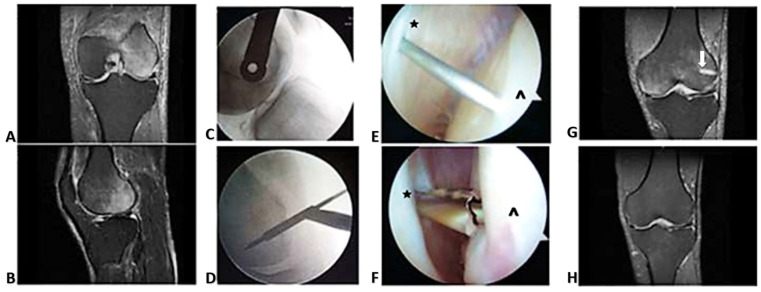
Migratory BME. (**A**) Coronal and (**B**) sagittal sections display high signal intensity in the lateral femoral condyle on the MRI T2 FS sequence. (**C**) Lateral and (**D**) AP X-ray views show fluoroscopic guidance of guide pin placement. (**E**,**F**) Arthroscopic guidance of pin and 4 mm cannulated drill tip through the joint capsule (*) to the femoral condyle (^). (**G**) BME observed in the medial femoral condyle on the coronal MRI T2 FS sequence. The previous decompression track is visible (arrow). (**H**) Coronal MRI T2 FS sequence showing resolution of high signal intensity in both condyles and healing of decompression tracks.

Case #2: A 53-year-old male with 3 months of spontaneous pain at the lateral femoral condyle. MRI revealed high signal intensity on T2 FS images at the lateral femoral condyle (Figure 2). He was treated with a surgical decompression that relieved his pain within 60 h. He presented again 5 weeks later, this time with spontaneous pain at the medial femoral condyle. MRI showed the characteristic high signal intensity in the medial femoral condyle on T2 FS images. The decompression track could be seen in the lateral femoral condyle together with incompletely resolved BME from his first treatment. A decompression was carried out in the medial femoral condyle with resolution of pain within a week. Post-treatment MRI at 12 weeks demonstrated resolution of BME in both condyles and no evidence of subchondral bone fracture. He remained asymptomatic with normal knee function at 1 year postoperative.

## 3. Discussion

The pathophysiological rationale for surgical decompression of BME is drawn from diverse observations of venous stasis, intraosseous hypertension, reduced perfusion, ischemia, and pain in BME and related conditions.

**Figure 3:**
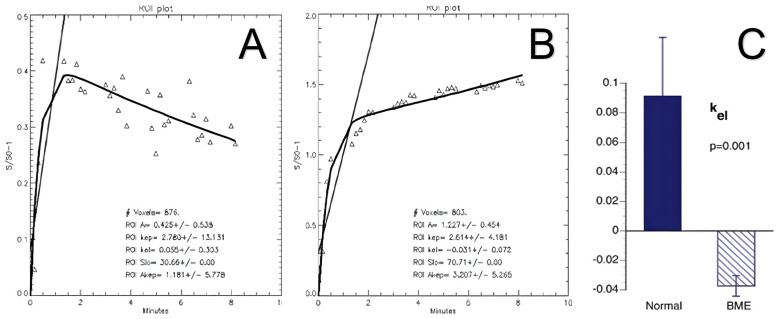
MRI time–intensity curves displaying inflow and outflow of Gd contrast agent. Gd concentration is proportional to blood flow and reflects perfusion. (**A**) In normal bone, outflow begins at 2 min of scan time and is completed within 10 min. (**B**) In BME, concentration continues to rise as outflow is obstructed (venous stasis). Pharmacokinetic modeling with the Brix equation allows quantification of the flow curve characteristics through derivation of perfusion constants. (**C**) k_el_ represents the contrast elimination constant and reveals quantitative differences in venous outflow between normal bone and BME. Adapted with permission from Ref. [16]. 2007, *Annals of the New York Academy of Sciences*.

Several studies have suggested that the pain and high signal intensity on T2 FS MRI of BME are most likely caused by venous outflow obstruction leading to elevated intraosseous pressure (IOP) or intraosseous hypertension [5]. With time, the IOP rises to the point that arterial inflow can be compromised, leading to intraosseous ischemia. Venous stasis and outflow obstruction in BME of various associations were initially demonstrated by static imaging with contrast venography [12,13,14]. More recently, a study using dynamic gadolinium (Gd)-enhanced MR imaging demonstrated venous stasis associated with BME (Figure 3). Washout of Gd from normal knees proceeded by 5 min and was complete by 10 min after contrast administration. In BME lesions, Gd washout and signal enhancement were delayed beyond 20 min of scan time [1]. Pharmacokinetic modeling enables the extraction of quantitative dynamic parameters of perfusion and has confirmed venous stasis with secondary reduced perfusion in human subjects. These and other studies with dynamic imaging have shown that spontaneous BME, as well as BME associated with OA and AVN, are accompanied by venous outflow obstruction and stasis [15].

A functional study of spontaneous BME revealed pathologically high intramedullary pressures with a mean of 73 mm Hg (range 50–90 mm Hg) and related the IOP to venous stasis [17]. In another study, intraosseous hypertension was found on pressure measurements in BME [18]. Surgical fenestration into cancellous bone abolishes both intraosseous hypertension and bone pain and increases pO2 [13,14,19]. The resolution of both the clinical symptoms and the MRI appearance by surgical decompression lends support to the hypothesis that the venous stasis in bone is causally related to the clinical syndrome of BME and its imaging manifestations.

Venous stasis has been associated with elevated IOP in several clinical conditions and has been associated with bone pain, especially at the knee [12,20,21,22,23]. A close relationship has been described between intraosseous hypertension and bone pain independent of the presence or absence of OA. Patients with bone pain at rest exhibited an IOP > 40 mmHg, while those with IOP < 35 mmHg did not [22]. Venous stasis, intraosseous hypertension, perfusion, and ischemia are mechanistically related. Linear relationships have been described between IOP and perfusion [24]. An elevation in IOP from 26–45 mm Hg reduces bone perfusion by 60%, while reduced bone perfusion results in intraosseous ischemia. Venous outflow obstruction has been shown to reduce bone pO2 by a factor of 1.5 within 30 min of venous occlusion [25]. Ischemia, if sustained and severe, can result in marrow cell death, as is seen in OA and AVN [26,27].

There is no consensus on the treatment of BME. However, based upon the observed pathophysiology of venous stasis, infusion of the vasoactive prostacyclin analogue; iloprost; or surgical decompression, or forage, have been used. Iloprost has been used for reduction of pressure in pulmonary hypertension. While iloprost infusion has been reported to be successful for treating BME [28], it involves prolonged intravenous infusion over several days and presents complications of vasodilation and hypotension [17,29,30]. Proponents of iloprost infusion point to complications of surgical decompression, including prolonged protected weight bearing and fracture [31]. The cases presented here demonstrate the efficacy of surgical decompression of spontaneous BME of the knee with rapid return to function without protected weight bearing. Lastly, a 2022 systematic review comparing BME treatment modalities found that surgical decompression resulted in virtually equivalent pain resolution compared to iloprost infusion in studies examining outcomes 1–3 months postoperatively [32]. Other promising treatment modalities include non-steroidal anti-inflammatory drugs (NSAIDS), bisphosphonates, denosumab, and extracorporeal shockwave therapy (ESWT) [32].

Several small series of patients with spontaneous BME of the hip or knee treated with surgical decompression have shown pain relief within 7 days postoperative and resolution of high signal intensity on T2 FS MRI within 3–6 months depending upon follow-up times [33,34,35]. In our experience, as well as in the aforementioned studies, surgical decompression results in prompt and complete pain relief of spontaneous BME with minimal interference with function and no complications. Surgically related fractures can be prevented by placement of the core track in cancellous bone, avoiding corticocancellous transition zones. It has substantial advantages over iloprost infusion in that vasomotor instability does not occur and the time course to recovery is faster. We recommend that surgical decompression be considered in the setting of spontaneous BME accompanied by disabling prolonged pain without structural changes. As described here, the procedure has the potential to relieve pain without the prolonged management of other conservative treatment methods.

## 4. Conclusions

Observations of skeletal perfusion in spontaneous BME and related conditions suggest that the syndrome is causally related to venous stasis and intraosseous hypertension, and that these perfusion changes may result in ischemia. It is not our suggestion that all cases of BME need treatment, since some will resolve spontaneously. However, spontaneous BME accompanied by disabling pain of 2–3 months duration is ameliorated within a few days by surgical decompression as demonstrated in the cases presented here. It must be acknowledged that these clinical observations, however supported by physiological rationale, are uncontrolled, and a randomized controlled trial is needed to examine the surgical hypothesis contained herein. There is no consensus treatment of BME other than treatment of consequential conditions when they occur, often late in the course when structural damage to the subchondral bone has occurred.

## Data Availability

Not applicable.
